# Variation of free‐energy landscape of the p53 C‐terminal domain induced by acetylation: Enhanced conformational sampling

**DOI:** 10.1002/jcc.24494

**Published:** 2016-10-13

**Authors:** Shinji Iida, Tadaaki Mashimo, Takashi Kurosawa, Hironobu Hojo, Hiroya Muta, Yuji Goto, Yoshifumi Fukunishi, Haruki Nakamura, Junichi Higo

**Affiliations:** ^1^ Institute for Protein Research Osaka University, 3‐2 YamadaokaSuitaOsaka565‐0871Japan; ^2^ Technology Research Association for Next Generation Natural Products Chemistry, 2‐3‐26 AomiKoto‐KuTokyo135‐0064Japan; ^3^ IMSBIO Co, Ltd, Owl Tower 6F, 4‐21‐1Higashi‐ikebukuro, Toshima‐kuTokyo170‐0013Japan; ^4^ Hitachi Solutions East Japan, 21‐1 EkimaehonchoKawasaki‐kuKanagawa210‐0007Japan; ^5^ Molecular Profiling Research Center for Drug Discovery (molprof), National Institute of Advanced Industrial Science and Technology (AIST), 2‐3‐36, AomiKoto‐kuTokyo135‐0064Japan

**Keywords:** free energy landscape, p53 C‐terminal, intrinsically disordered, post‐translation modification, multicanonical

## Abstract

The C‐terminal domain (CTD) of tumor suppressor protein p53 is an intrinsically disordered region that binds to various partner proteins, where lysine of CTD is acetylated/nonacetylated and histidine neutralized/non‐neutralized. Because of the flexibility of the unbound CTD, a free‐energy landscape (FEL) is a useful quantity for determining its statistical properties. We conducted enhanced conformational sampling of CTD in the unbound state via virtual system coupled multicanonical molecular dynamics, in which the lysine was acetylated or nonacetylated and histidine was charged or neutralized. The fragments were expressed by an all‐atom model and were immersed in an explicit solvent. The acetylation and charge‐neutralization varied FEL greatly, which might be convenient to exert a hub property. The acetylation slightly enhanced alpha‐helix structures that are more compact than sheet/loop conformations. The charge‐neutralization produced hairpins. Additionally, circular dichroism experiments confirmed the computational results. We propose possible binding mechanisms of CTD to partners by investigating FEL. © 2016 The Authors. Journal of Computational Chemistry Published by Wiley Periodicals, Inc.

## Introduction

An intrinsically disordered region (IDR) of a protein is highly flexible in physiological conditions unless interacting with its partner molecule.^1^ Structurally ordered proteins are well known to exert their biological functions through well‐defined quaternary, tertiary, and secondary structures. In contrast, IDRs exert their functions actively using conformational flexibility. Signal transduction is a typical function of IDR,^2^ where a single IDR interacts with different partner molecules to regulate the signal transduction. This multipartner interaction property is called a *hub* property.^3^ IDRs are related to some diseases such as cancer, diabetes, and neurodegenerative disease.^4^ Therefore, IDRs are an important subject related to biology, biophysics, and medical science.

Actually, IDRs can be characterized as (1) dynamic conformations (conformational ensemble), (2) post‐translation modification (PTM), and (3) roles of IDR–partner interaction. (1) An IDR does not adopt a specific tertiary structure in the unbound state. Consequently, IDR is not characterized by a single tertiary structure but by a conformational ensemble.^5^ The relation between the conformational ensemble and its biological functions has not been understood sufficiently.^6^ Presumably, the conformational ensemble architecture affects the binding mechanism. (2) PTM is found frequently in IDR. Importantly, PTM such as methylation, phosphorylation, and acetylation alters the physicochemical properties of IDR and modulates the functionality.^2^ Consequently, it is interesting to investigate the variation of the conformational ensemble by PTM. (3) Some IDRs adopt specific tertiary structures when interacting with their partners. This phenomenon is known as *coupled folding and binding*.^7^ Two interaction mechanisms have been proposed for coupled folding and binding processes: induced folding^8^, ^9^ and conformational selection.^9^, ^10^ In induced folding, IDR binds to the partner with conformations differing from that adopted in the complex (i.e., genuine conformation found in the native complex form). Subsequently, the IDR conformation varies until reaching the most stable complex structure. In conformational selection, the genuine bound conformation is involved in advance in the conformational ensemble of the unbound state. This bound form is used to bind to the partner. Recently, a computational report has described that coupled folding and binding takes place by a combination of the induced folding and the conformational selection.^11^ Another IDR–partner interaction picture is fuzzy‐complex formation, by which IDR binds to its partner adopting multiple conformations.^12^ Therefore, in this mechanism, conformational disorder/flexibility emerges in both bound and unbound states. Enthalpic stability for the individual bound form is less important for discussing the complex formation. A fly casting mechanism provides an alternative viewpoint to the IDR–partner interaction scheme.^13^ An IDR has a greater interaction radius than that of a folded protein. It captures partners at a large distance like a fishing line.

Tumor suppressor protein p53 consists of four functional domains: the transactivation domain (TAD) [residues 1–43], the DNA binding domain (DBD) [100–300], the tetramerization domain (TET) [320–360], and the C‐terminal negative regulatory domain (CTD) [363–393]. Both TAD and CTD are IDRs that interact with many molecules. These domains possess a hub property.^14^ To date, three TAD‐partner complex structures have been solved, where TAD adopts helical structures on surfaces of the partners in all three cases.^15^, ^16^, ^17^ Four CTD‐partner complex structures have been determined, where CTD adopts various structures: a helical structure to bind to S100B (PDB ID: 1DT7),^18^ a sheet to Sir2 (PDB ID: 1MA3),^19^ and fuzzy/coiled structures to CBP (PDB ID: 1JSP),^20^ and Cyclin A (PDB ID: 1H26).^21^ It is particularly interesting that CTD uses a *common sequence* [380–386] to bind to these four partners. In this sense, this short common binding region has the hub property. Furthermore, the states of histidine 380 (H380) and lysine 382 (K382) in CTD vary depending on the binding partner as follows: when binding to S100B, K382 is acetylated, although H380 can take either a positively charged or neutralized state depending on the pH condition.^18^, ^22^ To Cyclin A, K382 is nonacetylated and H380 is neutralized.^21^ To Sir2, K382 is acetylated and H380 is positively charged.^19^ To CBP, K382 is acetylated and H380 is neutralized.^20^ The correspondence between the state of CTD and the partner is presented in Table 1.

**Table 1:** Binding partners of CTD fragments.

	CTD fragment			
Partner	NonAc(H^+^)	Ac(H^+^)	NonAc	Ac
S100B^[a]^	P^[e]^	N	P	N
Cyclin A^[b]^	N^[f]^	N	P	N
Sir2^[c]^	N	P	N	N
CBP^[d]^	N	N	N	P

[a] Complex structures were referred from another report.^18^ Binding affinity of CTD to S100B was determined from an *in vitro* experiment.^22^

[b] Complex structures were referred from another report.^21^

[c] Complex structures were referred from another report.^19^

[d] Complex structures were referred from another report.^20^

[e] Mark “P” means that CTD can bind to the partner.

[f] Mark “N” means that CTD cannot bind to the partner.

Actually, CTD and its partner have been studied using molecular simulations: Allen et al. demonstrated trends of fluctuations around CTD binding sites of partners.^23^ Chen et al. assessed which population sellection or the induced folding is plausible to bind to S100B using molecular dynamics (MD) with an implicit solvent model and some simplifications of the protein model.^24^ McDowell et al. showed heterogeneity of CTD and the binding mechanism of CTD to S100B.^25^ Staneva et al. investigated conformational preferences of the unbound CTD using an implicit‐water Monte Carlo simulation.^26^ Although these studies provided beneficial knowledge for the conformational ensemble of CTD, they did not clarify the effects of acetylation on the CTD's conformational ensemble. We consider that computation of a free‐energy landscape is crucially important to elucidate the effects of acetylation on the conformational ensemble. To that end, a powerful conformational sampling method is necessary.

A multicanonical simulation has been introduced to enhance the sampling of complicated systems.^27^, ^28^, ^29^ Nakajima et al. introduced a multicanonical MD simulation (McMD) using Cartesian coordinates for dynamic variables. Adoption of Cartesian coordinates produced a multimolecular system that is tractable without special devices in a computer program. McMD generates various conformations under equilibrium conditions, which provides not only the most thermodynamically stable state but also intermediate states of the system. Importantly, a free‐energy landscape at arbitrary temperature is computable from the resultant conformational ensemble. To increase the sampling efficiency of McMD, trivial trajectory parallelization McMD (TTP‐McMD) was developed,^30^, ^31^ and applied to systems consisting of an intrinsically disordered segment and its partner protein.^11^, ^32^ Recently, a virtual system coupled McMD (V‐McMD) was also developed to increase the McMD sampling efficiency.^33^ Terakawa et al. performed all‐atom TTP‐V‐McMD of a p53 linker region (40 residues long) to design force‐field parameters for a coarse‐grained simulation model. The study reproduced an X‐ray scattering profile using the force field.^34^

For this study, we examined four CTD fragments in an unbound state (i.e., single‐chain state) using TTP‐V‐McMD, where K382 was nonacetylated or acetylated and H380 was positively charged or neutralized. The system was treated with an all‐atom model in an explicit solvent. The free‐energy landscapes were computed from the sampled conformations. Results show that the free‐energy landscape of the single‐chain state varies by the K382 acetylation and the H380 neutralization. In fact, the IDR‐partner binding is controlled not only by the finally formed complex structure but also by the conformational distribution of the unbound state. We suggest possible binding mechanisms of CTD to their partner molecules, S100B, Sir2, CBP, and Cyclin A with investigation of the single‐chain free‐energy landscapes.

## Theory

In a conventional (canonical) MD simulation of a biomolecular system, the force acting on an atom i is computed as $f_{i}=-{\text{grad}}_{i}E_{R}(r)$, where $E_{R}(r)$ is a potential energy of the system as a function of the coordinates of the constituent atoms $r=[r_{1},r_{2},\ldots ,r_{N\text{atom}}]$, and where $N_{\text{atom}}$ is the number of atoms (polypeptide atoms plus solvent atoms) and $r_{i}=[x_{i},y_{i},z_{i}]$ is the position vector of atom i, that is, the *x*, *y*, and *z* coordinates. An energy distribution obtained from the canonical MD converges to the following canonical distribution as

(1) $$P_{c}(E_{R},T)=Z_{c}{}^{-1}n_{R}(E_{R})\text{exp}\left[-\frac{E_{R}}{R_{\text{gas}}T}\right],$$

where $R_{\text{gas}}$ represents the gas constant, $n_{R}(E_{R})$ denotes the density of states (DOS) of the system at the potential energy $E_{R}$, T stands for temperature, and $Z_{c}=\int n_{R}(E_{R})\text{exp}\left[-E_{R}/R_{\text{gas}}T\right]\;dE_{R}$ represents a partition function (i.e., a normalization factor) at T.

McMD uses a modified potential energy $E_{\text{mc}}$ instead of $E_{R}$, which is defined formally as

(2) $$\begin{array}{c} E_{\text{mc}}(E_{R})=E_{R}+R_{\text{gas}}T\text{ln}\left[P_{c}(E_{R},T)\right]\\ \;\;\;\;=R_{\text{gas}}T\text{ln}\left[n_{R}(E_{R})\right]. \end{array}$$

The force acting on the atom *i* is represented as

(3) $$f_{i}{}^{\text{mc}}=-{\text{grad}}_{i}E_{\text{mc}}.$$

An energy distribution $P_{\text{mc}}(E_{R})$ from the McMD simulation at *T* is given simply by replacing $E_{R}$ by $E_{\text{mc}}$ in eq. (1) as

(4) $$\begin{array}{c} P_{\text{mc}}(E_{R})=Z^{-1}{}_{\text{mc}}(T)n_{R}(E_{R})\text{exp}\left[-\frac{E_{\text{mc}}}{R_{\text{gas}}T}\right]\\ =Z^{-1}{}_{\text{mc}}(T)\frac{n_{R}(E_{R})}{n_{R}(E_{R})}\\ =\text{const}, \end{array}$$

where $Z_{\text{mc}}=\int n(E_{R})\text{exp}\left[-E_{\text{mc}}/R_{\text{gas}}T\right]\;dE_{R}$ is a partition function at *T*. This equation guarantees that the energy distribution from a longtime McMD converges to a flat function. Then, the flatness of *P*_mc_(*E*_R_) is a measure to judge the convergence of sampling.

Virtual system coupled McMD (V‐McMD) has been developed to increase the sampling efficiency of McMD, where a “virtual system” is introduced with setting its physical quantities arbitrarily.^33^ From here, we explain the framework of V‐McMD: Assume that a virtual system exists in addition to the real system (i.e., the biomolecular system). We define an entire system as the sum of the real system and the virtual system. Here we assume that the virtual system does not interact explicitly with the real system. In other words, no cross‐term exists in the potential energy of the entire system. Then, the total potential energy is given as $E_{\text{tot}}=E_{R}+E_{v}$, where $E_{v}$ is the virtual system's potential energy, a function of a virtual system's coordinate *v*. Accordingly, DOS of the entire system is represented as $n_{\text{tot}}(E_{R},E_{v})=n_{R}(E_{R})n_{v}(E_{v})$, where $n_{v}$ is DOS of the virtual system. Using the arbitrary property of the virtual system, we simply set this DOS as $n_{v}(E_{v})=1$. Then, the multicanonical energy $E_{\text{vmc}}$ of the entire system is given as

(5) $$\begin{array}{c} E_{\text{vmc}}=R_{\text{gas}}T\text{ln}\left[n_{\text{tot}}(E_{R},E_{v})\right]\\ \;\;=R_{\text{gas}}T\text{ln}\left[n_{R}(E_{R})\right]. \end{array}$$

From eq. (5), the energy distribution is calculated as

(6) Pvmc(ER,Ev)=Z−1vmc(T)ntot(ER,Ev)exp[−EvmcRgasT]      =Z−1vmc(T)nR(ER)nR(ER)      =const,

where $Z_{\text{vmc}}=\int n_{\text{tot}}(E_{R},E_{v})\text{exp}[-E_{\text{vmc}}/R_{\text{gas}}T]dE_{R}dE_{v}$ is a partition function of the entire system at *T*. As with eqs. (4) and (6) also ensures that the two‐dimensional distribution $P_{\text{vmc}}(E_{R},E_{v})$ converges to an even function after a long V‐McMD simulation.

Generally, it is more difficult to achieve convergence of the two‐dimensional distribution than that of one‐dimensional one. The virtual system can be set arbitrarily. Then, we discretize coordinate *v* to reduce the sampling space as $v=v_{i}$, where $i=1,\ldots ,n_{\text{vs}}$ and $n_{\text{vs}}$ is the number of the virtual states allowed. We introduce the term “virtual state” to specify the state of the discretized virtual system. The *i*th virtual state corresponds to $v_{i}$. Accordingly, $E_{v}$ is discretized as $E_{vi}$. Therefore, the time‐evolution of the virtual system is done using the Metropolis Monte Carlo scheme.

**Figure 1:**
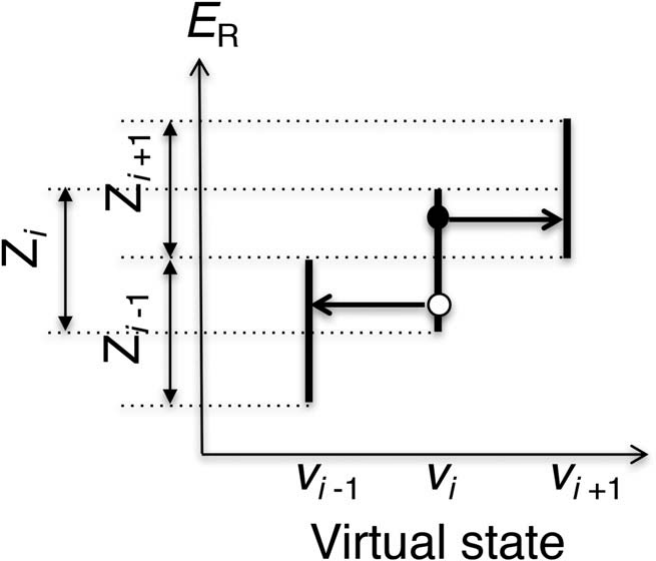
Transitions among adjacent virtual states. Presuming that the system is at the filled‐circle position, where potential energy is $E_{R}$ and the virtual state belongs to $v_{i}$, then, the virtual state might jump to $v_{i+1}$ without changing atomic positions (i.e., without changing $E_{R}$). Its transition probability is given in eq. (7). However, when the system is at the open‐circle position, the virtual state might transition to $v_{i-1}$.

Now, the system is specified by two quantities: $E_{R}$ and $v_{i}$. We set the action of the virtual system on the biomolecular system so that $E_{R}$ is confined in a zone $Z_{i}=\left[E_{R}{}^{\text{min}}(v_{i})<E_{R}<E_{R}{}^{\text{max}}(v_{i})\right]$ when its virtual state is in $v_{i}$. The method for the confinement is explained in an earlier report^33^. We set the zones so that $Z_{i}$ overlaps with $Z_{i-1}$ and $Z_{i+1}$, although $Z_{i-1}$ and $Z_{i+1}$ have no mutual overlap as presented in Figure 1. The actually used zones are given later. Assuming that the system is at the filled‐circle position of Figure 1, for which energy $E_{R}$ is involved in $Z_{i}$ in the virtual state $v_{i}$, then, this potential energy is also involved in $Z_{i+1}$. Therefore, the state $v_{i}$ can transition to $v_{i+1}$ using the Monte Carlo method. The molecular configuration r is not changed (i.e., $E_{R}$ is not altered) in this transition. The transition from $v_{i}$ to $v_{i-1}$ might occur because $E_{R}$ is involved in $Z_{i}$ and $Z_{i-1}$ if the system is at the open‐circle position of Figure 1. We set the transition probability between virtual states $v_{i}$ and $v_{i\pm 1}$ as shown below.

(7) $$P_{t}(E_{R},v_{i}\to v_{i\pm 1})=\{\begin{array}{c} 1\;\;(E_{R}\in Z_{i\pm 1})\\ 0\;\;(\text{otherwise}) \end{array}$$

Equation (7) establishes that the transition occurs unconditionally. However, the probability can be set arbitrarily in general. We operate the virtual‐state transition once every $N_{\text{int}}$ steps. Then, r moves according to MD scheme from the *i*th to the $\left(i+N_{\text{int}}\right)$ th steps without changing the virtual state $v_{i}$. Consequently, $v_{i}$ might transition to $v_{i-1}$ or $v_{i+1}$ at the $\left(i+N_{\text{int}}\right)$ th step without changing r. The actual value for the transition interval $N_{\text{int}}$ is given later. Because of eq. (7), the energy distribution for each virtual state converges to a constant.

(8) $$P_{\text{vmc}}(E_{R},v_{i})=\{\begin{array}{c} \text{const}\;\;\left(\text{in}\;\text{zone}\;Z_{i}\right)\\ 0\;\;\left(\text{outside}\;\text{of}\;Z_{i}\right) \end{array}$$

## Materials and Methods

We constructed four fragment systems of p53 CTD, all of which consist of 17 amino‐acid residues (residues 372–388 in UniProt^35^) and amino acid sequences in single‐letter codes as (i) Ace‐KKGQSTSR‐H ^+^ ‐KKLMFKTE‐NH2, (ii) Ace‐KKGQSTSR‐H ^+^ ‐K‐aK‐LMFKTE‐NH2, (iii) Ace‐KKGQSTSRHKKLMFKTE‐NH2, and (iv) Ace‐KKGQSTSRHK‐aK‐LMFKTE‐NH2, where aK, H^+^, Ace, and NH2, respectively, represent acetylated lysine, positively charged histidine, acetyl cap, and amine cap. As described in this article, we refer, respectively, to these fragments as (i) NonAc(H^+^), (ii) Ac(H^+^), (iii) NonAc, and (iv) Ac. These fragments include in common the binding regions (residues 380–386) to the four partners S100B, Sir2, CBP, and Cyclin A. We designate the regions as “common binding regions” in this study, which are shown as underlined in the sequences above. Remember that Table 1 presents correspondence between the fragment and its binding partner molecule.

We put each of the fragments in a periodic box (50 × 50 × 50 Å^3^) filled with water molecules and ions, where the number of ions is set so that the net charge of the whole system is zero and the ionic concentration is set to a physiological salt one. The conformations of the CTD fragments were the helical structure taken from the S100B‐CTD complex. As explained later, these helical structures were randomized quickly in a preparative canonical MD simulation at a high temperature. Table 2 presents characteristics of the simulation systems.

**Table 2:** Simulation systems.

	System			
	NonAc(H^+^)	Ac(H^+^)	NonAc	Ac
Total atoms	12,196	12,196	12,197	12,197
Water molecules	3954	3953	3955	3954
Ions	Na: 11, Cl: 17	Na: 11, Cl: 16	Na: 11, Cl: 16	Na: 11, Cl: 15
Box size^[a]^	(49.3)^3^	(49.2)^3^	(49.1)^3^	(49.2)^3^

[a] Periodic box size is given in Å^3^ unit. Shown values are those obtained after NPT simulation at 300 K and 1 atm.

Before V‐McMD simulations, we performed a constant‐pressure (NPT) simulation at 300 K and 1 atm to ascertain the periodic box size for each system. The resultant box sizes are presented in Table 2. Then, we conducted a long high‐temperature (600 K) constant‐volume (NVT) simulation (time step: 2.0 fs) to randomize the helical conformation of each system.

Generally, DOS ( $n_{R}(E_{R})$) is required to perform multicanonical sampling [see eq. (2)]. However, DOS is unknown *a priori*. Consequently, to begin with, we approximated DOS by performing conventional MD runs (i.e., canonical MD runs) at different temperatures covering a wide temperature range [280 K–600 K]. Then, as reported in the literature,^33^ the resultant canonical energy distributions at the various temperatures are integrated to approximate DOS, which is used to define $E_{\text{vmc}}$ for the first V‐McMD simulations [see eq. (5)]. DOS was estimated in the range of 280 K to 600 K. Therefore, the subsequently performed V‐McMD simulations aim to obtain a flat energy distribution [eq. (6)] in this range. The upper temperature limit (600 K) was set so that the fragment overcame various energy barriers. The *Results and Discussion* section shows that various conformations were sampled. The lower limit (280 K) was lower than a room temperature (300 K). Therefore, the obtained conformational ensemble involved conformations probable at 300 K.

The first V‐McMD simulation was performed with using $E_{\text{vmc}}(=E_{\text{mc}})$ obtained above, where 32 runs were executed in parallel starting from the randomized conformations sampled from the high‐temperature simulation. Therefore, we used the TTP procedure to perform V‐McMD,^31^, ^32^ although we do not explicitly use the term “TTP‐V‐McMD” in this article. After the first V‐McMD simulation, we updated $E_{\text{mc}}$ according to the method presented in an earlier report,^33^ and performed the second V‐McMD simulation with using the updated $E_{\text{mc}}$, and so on. The initial conformation for one of the 32 runs in the *i*th iteration is the last snapshot for the run in the (i−1) th iteration. We repeated this iteration procedure until the energy distribution converges to a function flat sufficient: $P_{\text{vmc}}\approx \text{const}$. Then, the final iteration is the production run to collect snapshots for analyses. The numbers of iterations were 8 for NonAc(H^+^), 9 for Ac(H^+^), 8 for NonAc, and 8 for Ac, where the length of the production run was 320 ns for all the systems. Table S1 of Supporting Information presents the simulation lengths, inter‐virtual state transition interval $N_{int}$, and virtual‐state zones $Z_{i}$ for each of iterations. One might consider that the simulation length of 320 ns for the production run is too short to obtain a statistically significant ensemble. However, the enhanced sampling method has higher efficiency than conventional sampling does. Later, we discuss statistical properties of the resultant ensembles.

We used a computer program psygene–G^36^ for V‐McMD with the SHAKE^37^ method to fix the covalent‐bond length related to hydrogen atoms, the zero dipole summation method^38^, ^39^, ^40^ to calculate long‐range electrostatic interactions, the velocity scaling method^41^ to control temperature, TIP3P model^42^ for water molecule, and an Amber‐based hybrid force field for p53 CTD.^43^ The Amber‐based hybrid force field is defined as $E_{\text{hybrid}}(\omega )=(1-\omega )E_{94}+\omega E_{96}$, where $E_{94}$ and $E_{96}$_,_ respectively, denote param 94 and param 96 AMBER force fields^44^, ^45^, and where ω (0≤ω≤1) is a mixture weight for $E_{94}$ and $E_{96}$. Kamiya et al. confirmed that a proper range for ω is 0.45≤ω≤0.95.^43^ Ikebe et al. showed that the larger the value of ω, the smaller the helical propensity in the resultant conformational ensemble at 300 K.^46^ In our previous simulation of an IDP system,^11^ we set ω=0.75 and obtained results comparable to experimentally obtained results. However, in our preparative simulation of the present systems with setting ω=0.75, the resultant ensemble exhibited a considerably high helical content. Then, we set ω=0.80 for the current study. The MD time step was set to 2.0 fs for all the simulations.

We constructed the force‐field parameters of acetylated lysine (aK) as follows: The dihedral angle parameters are the same as those of lysine in the Amber force field. The atomic partial charges of aK were from a force field (Ref: http://pc164.materials.uoi.gr/dpapageo/amberparams.php).

### Conformational ensemble and free‐energy landscape

The V‐McMD simulation of each system produces a conformational ensemble that consists of snapshots of various energies. The statistical weight assigned to a snapshot of energy $E_{R}$ at temperature *T* is equivalent to the canonical energy distribution $P_{c}(E_{R},T)$ [eq. (1)]. We denote this ensemble as $Q_{\text{SYS}}$, where the notation “SYS” specifies the computed system as SYS = NonAc(H^+^), Ac(H^+^), NonAc, or Ac. Accordingly, the statistical weight of each system is denoted as $P_{c}^{\text{SYS}}(E_{R},T)$. The summation of the four ensembles is denoted as $Q_{\text{sum}}$: $Q_{\text{sum}}=\sum_{\text{SYS}}Q_{\text{SYS}}$. In this study, we set T=300 K to prepare $Q_{\text{SYS}}$, although we do not mention explicitly that the statistical weight is set at 300 K.

To analyze the conformational ensemble, we generate a two‐dimensional (2D) free‐energy landscape using principal component analysis (PCA) as follows: First, we compute inter‐C α atomic distances for each snapshot in $Q_{\text{sum}}$, and define a vector as $q=[q_{1},q_{2},\ldots ,q_{N\text{pair}}]$, where $q_{i}$ is a distance for a Cα atomic pair and $N_{\text{pair}}$ is the number of the pairs. Remember that the number of residues is 17 for all four systems. Consequently, $N_{\text{pair}}$ is 136 ( =17×(17−1)/2) for all systems. Then, we calculated a variance–covariance matrix A with elements (i,j) expressed as $A_{ij}=〈q_{i}q_{j}〉-〈q_{i}〉〈q_{j}〉$, where brackets are the ensemble average over conformations in $Q_{\text{sum}}$. Diagonalizing this matrix, we obtained $N_{\text{pair}}$ eigenvectors ( $v_{1},v_{2},\ldots ,v_{N\text{pair}}$) and eigenvalues ( $\lambda_{1},\lambda_{2},\ldots ,\lambda_{N\text{pair}}$), where $v_{i}$ and $\lambda_{i}$ are paired satisfying an equation $Av_{i}=\lambda_{i}v_{i}$. The eigenvectors satisfy an orthogonal and normalized relation: $v_{i}\cdot v_{j}=\delta_{ij}$. We presume that the eigenvalues are arranged in descending order as $\lambda_{1}>\lambda_{2}>\ldots$.

We use $v_{1}$ and $v_{2}$ to construct the 2D space (2D PCA space) by setting the coordinate axes to $v_{1}$ and $v_{2}$, and to generate a conformational distribution by projecting conformations in $Q_{\text{SYS}}$ to the 2D PCA space. The coordinate axis $v_{i}$ is designated as a principal component (PC) axis i. We denote the *k*th conformation in $Q_{\text{SYS}}$ as $q^{\left(k\right)}$ ( $=[q_{1}^{\left(k\right)},q_{2}^{\left(k\right)},\ldots ,q_{N}^{\left(k\right)}]$). Then, the projection of $q^{\left(k\right)}$ to the axis $v_{i}$ is done by a scalar product: $x_{\text{PC}\;i}^{\left(k\right)}=q^{\left(k\right)}\cdot v_{i}$ ( i=1,2). The position of $q^{\left(k\right)}$ in the 2D PCA space is given by 2D coordinates $\left[x_{\text{PC}1}^{\left(k\right)},x_{\text{PC}2}^{\left(k\right)}\right]$. Repeating this procedure for all conformations in $Q_{\text{SYS}}$, we obtain the distribution of conformations in the 2D PCA space: $P_{}^{\text{SYS}}(x_{\text{PC}1},x_{\text{PC}2},T)$, where conformation of energy $E_{R}$ contributes to the distribution with the weight $P_{c}^{\text{SYS}}(E_{R},T)$. Finally, the *potential of mean force* (PMF) is computed as $F_{}^{\text{SYS}}(x_{\text{PC}1},x_{\text{PC}2},T)\equiv -R_{\text{gas}}T\text{ln}[P_{}^{\text{SYS}}(x_{\text{PC}1},x_{\text{PC}2},T)]$ with spatial patterns that are called the free‐energy landscape (FEL) in this study. From comparison of the spatial patterns of FEL among the four systems, we can discuss the difference of the architecture of FEL among the systems.

The ratio of contribution from the PC component i to the whole standard deviation is expressed as

(9) $$rc_{i}=\frac{\lambda_{i}}{\sum_{i=1}^{N}\lambda_{i}}$$

In PCA, the larger the eigenvalue assigned to the PC components i becomes, the greater the contribution: ${\text{SD}}_{1}>{\text{SD}}_{2}>\ldots$. Therefore, the 2D PCA space constructed by $v_{1}$ and $v_{2}$ is suitable to overview the conformational distribution. The contribution ratio by the PC components 1 and 2 is given simply as $rc_{1}+rc_{2}$.

### Synthesis of the nonacetyl and acetyl CTD fragments

CTD fragment with nonacetyl lysine (K382) was synthesized by the 9‐fluorenylmethoxycarbonyl (Fmoc) method at a 0.05 mmol scale using a peptide synthesizer (Liberty Blue; CEM Corp., NC). After the completion of the peptide chain assembly, the obtained resin was treated with TFA containing 2.5% triisopropylsilane and 2.5% distilled water for 2 h. The crude peptide was concentrated by a nitrogen stream, precipitated by ether and purified by the reversed‐phase HPLC to obtain nonacetyl peptide. ESI mass, found: 1038.7, calcd. for [M + 2H]^2+^: 1038.8.

The synthesis of CTD fragment with acetyl‐lysine (aK382) was also performed by the synthesizer, except that Lys^11^ was introduced using Fmoc‐Lys(Aloc)‐OH (Aloc: allyloxycarbonyl), activated by *O*‐benzotriazol‐1‐yl‐*N*,*N*,*N*′,*N*′‐tetramethyluronium hexafluorophosphate (HBTU)/*N*,*N*‐diisopropylethylamine (DIEA). After the completion of the chain elongation, the solution of tetrakis(triphenylphosphine)palladium(0), phenylsilane, acetic anhydride in dichloromethane was added to the resin for 45 min to acetylate the side chain amino group of Lys selectively 11. The deprotection and purification were performed in the same manner to obtain Ac peptide. ESI mass, found: 1059.7, calcd. for [M + 2H]^2+^: 1059.8.

### Far‐UV circular dichroism measurements

The nonacetyl and acetyl CTD fragments were dissolved in Milli‐Q water at 200 μM. The peptide solutions were diluted to 40 μM with the desired concentration of trifluoroethanol (TFE) for circular dichroism (CD) measurements. To detect the helix propensity, we varied TFE concentrations of 0–50%. It is noteworthy that the TFE concentrations in this article are volume per volume percentages. The peptide solutions also contained 25 mM sodium phosphate (pH 7.0 without TFE) and 0.1 M sodium chloride. Because pH is 7.0, the histidine state is neutral. Consequently, the fragments treated in this CD measurement are also designated as NonAc and Ac.

We performed far‐UV CD measurements of the peptide solutions. Far‐UV CD spectra were recorded on a spectropolarimeter (JASCO J‐820; Jasco Corp., Japan) at 25°C using a quartz cuvette with 1‐mm path length. The spectra were expressed as the mean residue ellipticity and [θ] (deg cm^2^ dmol^−1^). All spectra were estimated iteratively 16 times. Furthermore, this procedure was repeated three times to compute the average and standard deviation of the spectrum.

## Results and Discussion

**Figure 2:**
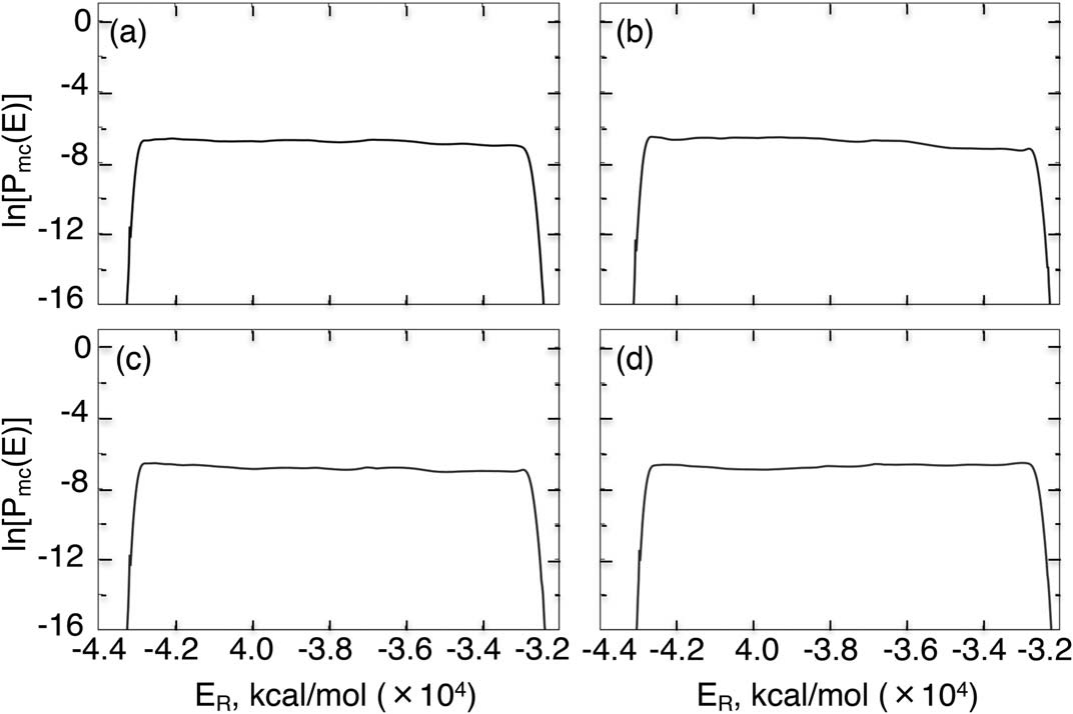
Flat energy distribution for the a) NonAc(H^+^), b) Ac(H^+^), c) NonAc, and d) Ac systems. Individual distributions ( $P_{\text{vmc}}(E_{R},v_{i})$, $i=1,\ldots ,n_{\text{vs}}$) for the virtual states are integrated into the shown distribution $P_{\text{mc}}(E_{R})$ using the method presented in an earlier report.^33^

The V‐McMD production run generated conformational ensemble $Q_{\text{SYS}}$ for the four CTD systems (SYS = NonAc(H^+^), Ac(H^+^), NonAc, and Ac) in the unbound state. Figure 2 demonstrates the flat distributions from the production runs. The flatness ensures that eq. (6) was satisfied (DOS was estimated accurately). Therefore, the sampling has been done with statistical significance. Below, we analyze effects of the acetylation of K382 and the charge neutralization of H380 on the free‐energy landscape and secondary structure contents. Furthermore, we discuss possible binding mechanisms of the four CTD fragments to their partner molecules. Last, we again check the statistical significance of the resultant ensemble by demonstrating the convergence of FEL.

### Free energy landscape of the full‐length fragments

**Figure 3:**
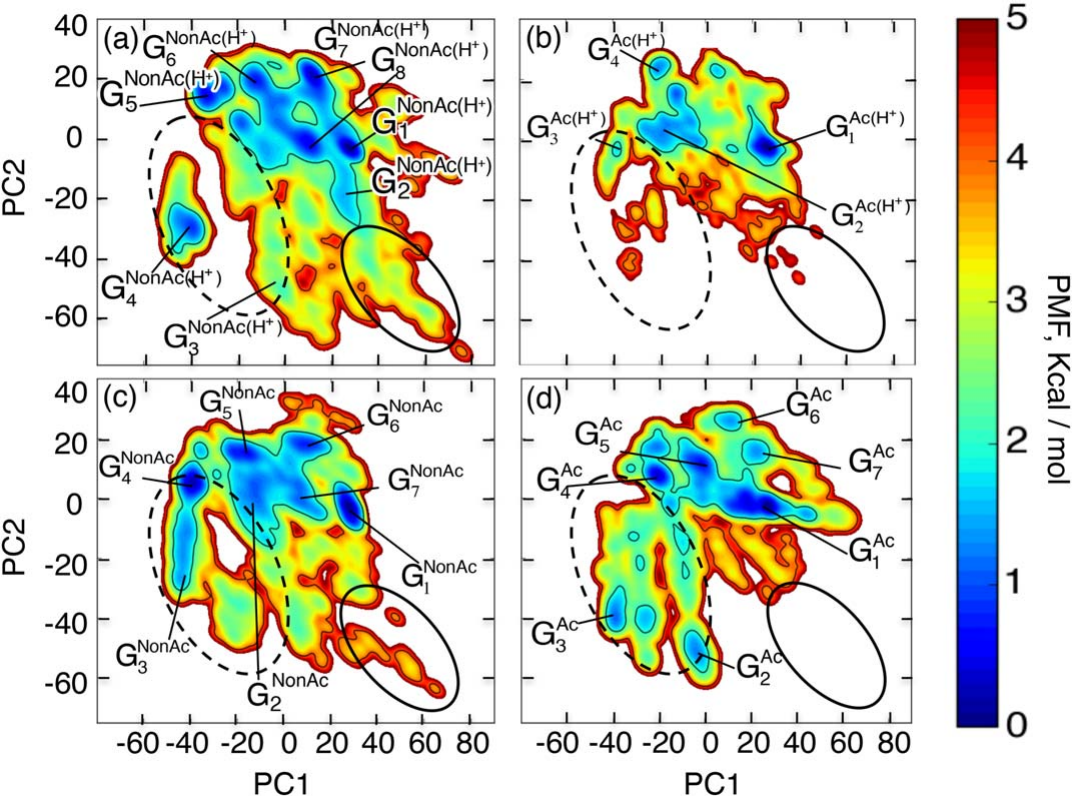
FEL, $F_{}^{\text{SYS}}$, at 300 K for entire (17‐residue) CTD fragments constructed in 2D PCA space: a) $F_{}^{\text{NonAc}(H+)}$, b) $F_{}^{\text{Ac}(H+)}$, c) $F_{}^{\text{NonAc}}$, and d) $F_{}^{\text{Ac}}$. We express a 2D site on *F* ^SYS^ as $r_{m}$. The lowest PMF site, $r_{\text{min}}$, is set as $F_{}^{\text{SYS}}(r_{\text{min}})=0.0$ kcal/mol. Clusters $G_{k}^{\text{SYS}}$ are shown in FEL. See main text for details of the naming. PCA1 and PCA2 axes are computed from the entire ensemble (i.e., $Q_{\text{sum}}$). Consequently, the PCA axes for all the panels are common. [Color figure can be viewed at wileyonlinelibrary.com]

**Figure 4:**
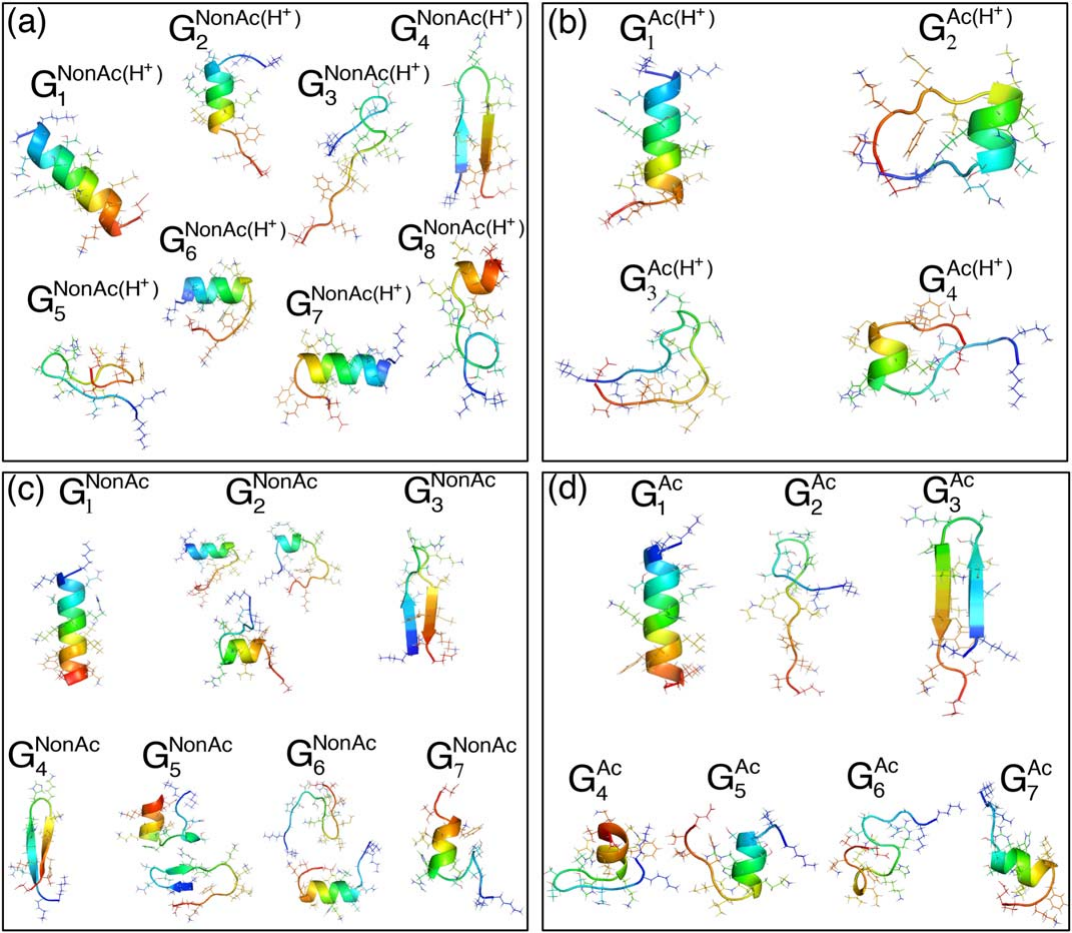
Structures in clusters $G_{k}^{\text{SYS}}$ of FEL for entire CTD fragments: Tertiary structures picked from clusters $G_{k}^{\text{SYS}}$ are colored by a rainbow (blue and red sides, respectively, show N and C‐termini). [Color figure can be viewed at wileyonlinelibrary.com]

Figure 3 shows FELs at 300 K constructed in the 2D PCA space. The contribution ratios $rc_{1}$ and $rc_{2}$ from $Q_{\text{sum}}$ were, respectively, 41.4% and 18.8%. Then, the contributions from the PC 1 and 2 axes ( $rc_{1}+rc_{2}$) were 60.2%. In Figure 3, we refer to the clusters as $G_{k}^{\text{SYS}}$, where superscript SYS specifies the computed system and the subscript k is a label assigned to the clusters. Figure 4 demonstrates representative tertiary structures in each cluster. In all panels, $G_{1}^{\text{SYS}}$ is assigned to the cluster of the global minimum PMF, which corresponds to a nearly complete helix (see structures in $G_{1}^{\text{SYS}}$ in Fig. 4) located at the same position in the 2D PCA space. Figure 3 manifests that the clusters can transition mutually at 300 K: the free‐energy barriers among the clusters are surmountable at 300 K, except for the cluster $G_{4}^{\text{NonAc}(H+)}$. In other words, the CTD fragments are disordered.

All FELs involved not only the complete‐helix cluster ( $G_{1}^{\text{SYS}}$) but also partially helical ones. The tertiary structures are shown in $G_{2}^{\text{NonAc}(H+)}$, $G_{6}^{\text{NonAc}(H+)}$, $G_{7}^{\text{NonAc}(H+)}$, and $G_{8}^{\text{NonAc}(H+)}$ in Figure 4a, in $G_{2}^{\text{Ac}(H+)}$ and $G_{4}^{\text{Ac}(H+)}$ in Figure 4b, in $G_{2}^{\text{NonAc}}$, $G_{5}^{\text{NonAc}}$, $G_{6}^{\text{NonAc}}$, and $G_{7}^{\text{NonAc}}$ in Figure 4c, and in $G_{4}^{\text{Ac}}$, $G_{5}^{\text{Ac}}$, and $G_{7}^{\text{Ac}}$ in Figure 4d. The β‐hairpins are also found as $G_{4}^{\text{NonAc}(H+)}$ in Figure 4a, as $G_{3}^{\text{Ac}(H+)}$ in Figure 4b (this is a distorted hairpin‐like structure), as $G_{3}^{\text{NonAc}}$, $G_{4}^{\text{NonAc}}$, and $G_{5}^{\text{NonAc}}$ in Figure 4c, and as $G_{3}^{\text{Ac}}$ in Figure 4d.

Although all the CTD fragments exhibited conformational diversity, the FEL shape is considerably different as shown in Figure 3. Remarkable differences are apparent for regions indicated by the solid‐line and broken‐line circles in the figure. Comparison of FELs between Figures 3a and 3b as well as between Figures 3c and 3d clarifies that the acetylation of K382 diminishes the probability in the region by a solid‐line circle. We find that extended conformations are distributed in the solid‐line circled region. It is likely that the acetylation facilitates hydrophobic‐core formation by making the CTD fragment compact, which results in disappearance of the extended conformations. To verify this expectation, we calculated a radius of gyration of the fragments at 300 K only using hydrophobic atoms in the CTD fragment: Cβ, Cγ, and Cδ atoms of K372, K373, K381, K382, and K386; Cβ atom of H380; Cβ atom of L383; Cβ atom of M384; and Cβ atom of P385. The side‐chain tip of the acetylated K382 in the Ac(H^+^) and Ac systems was excluded from the computation of radius of gyration for the strict comparison because the side‐chain tip does not exist in NonAc(H^+^) and NonAc. Table 3 presents the radius of gyration values (*R*g), which demonstrates that the acetylation induces a compact hydrophobic core. We discuss this point further by viewing the tertiary structures of the CTD fragments below.

**Table 3:** Radius of gyration computed from selected hydrophobic atoms at 300 K.

	NonAc(H^+^)	Ac(H^+^)
*R*g [Å]	8.6 ± 1.2	8.2 ± 0.8
	NonAc	Ac
*R*g [Å]	8.6 ± 1.0	8.1 ± 1.9

**Figure 5:**
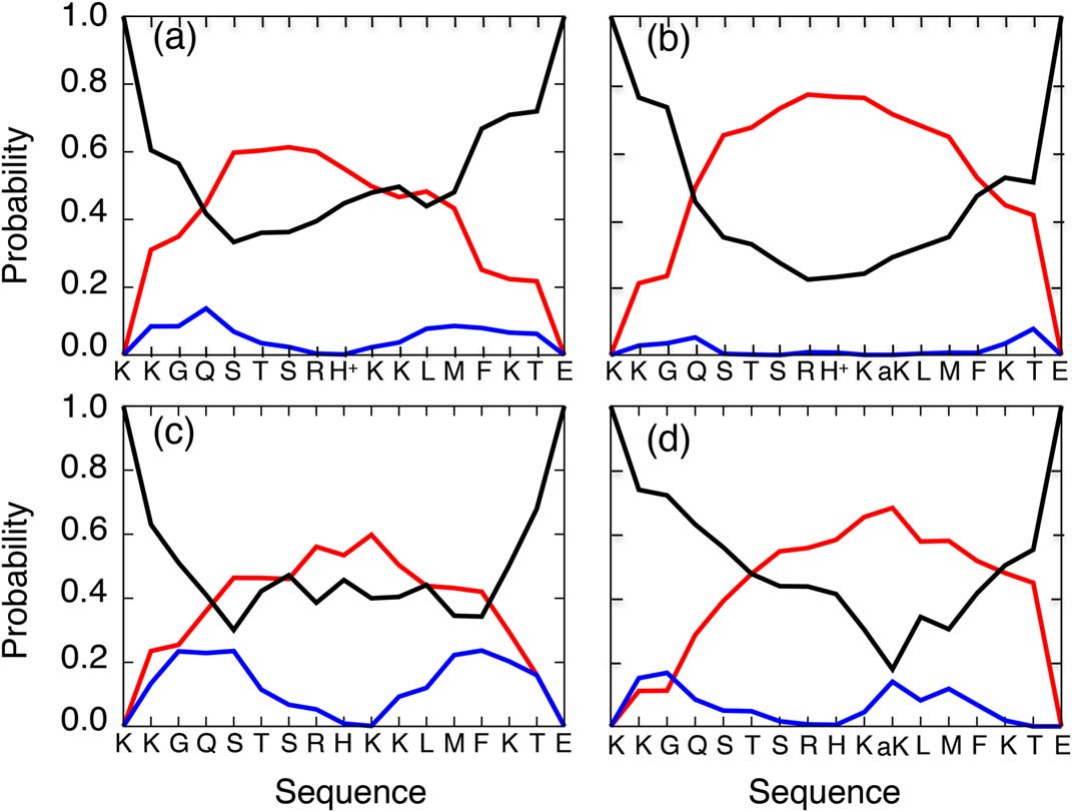
Secondary‐structure contents along CTD sequence at 300 K: (a) NonAc(H^+^), (b) Ac(H^+^), (c) NonAc, and (d) Ac. Secondary structures are classified into three types: “helix” for α‐helix, π‐helix, or 3‐10 helix; “sheet” for β‐bridge or extended β strand, and “other” for else. Red, blue, and black lines, respectively, present contents of helix, sheet, and other. [Color figure can be viewed at wileyonlinelibrary.com]

We computed the secondary‐structure propensity of each residue in $Q_{\text{SYS}}$ using the DSSP program.^47^ Figure 5 depicts the secondary‐structure contents along the sequence at 300 K. Comparison of Figures 5a and 5b reveals that K382 acetylation induces the helix propensity of the CTD fragment. This tendency is also apparent from comparison of Figures 5c and 5d. Furthermore, Table 4 presents the helix increment by the acetylation quantitatively.

**Table 4:** Secondary‐structure contents at 300 K for entire (17‐residue) CTD fragments.

	Helix (%)	Sheet (%)	Other (%)
NonAc(H^+^)	39.1	5.1	55.8
Ac(H^+^)	51.1	1.6	47.3
NonAc	36.3	12.4	51.2
Ac	41.2	5.9	53.0

**Figure 6:**
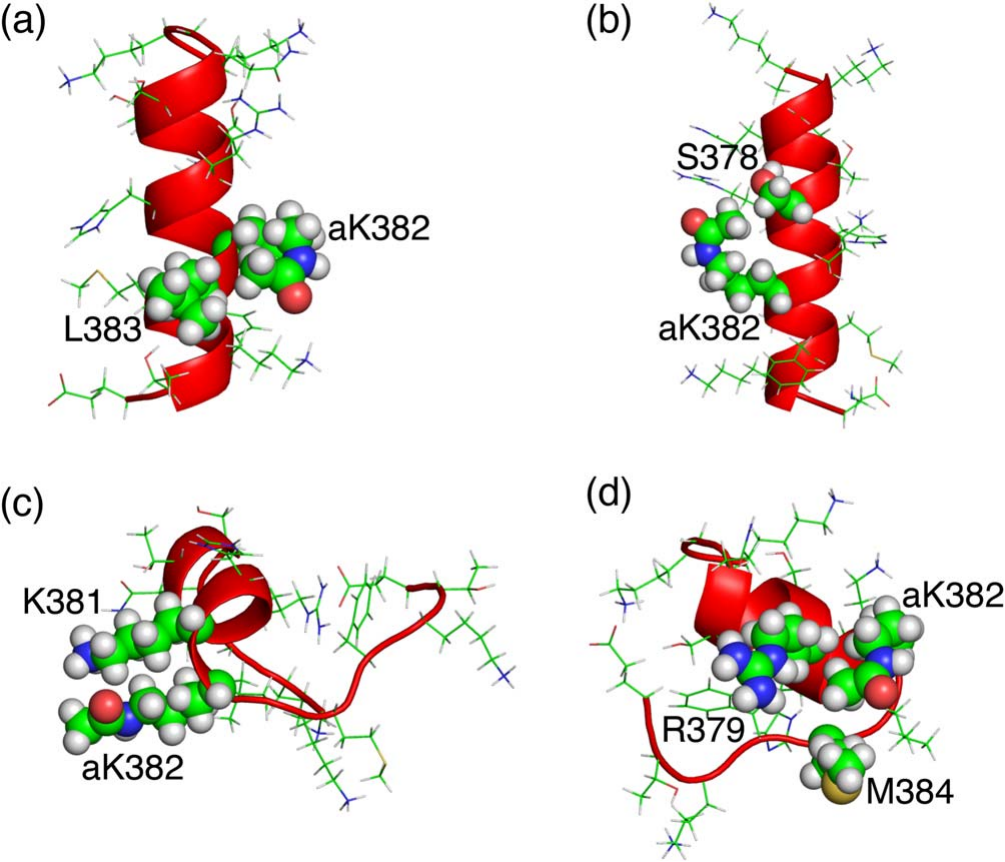
Tertiary structures taken from clusters a) $G_{1}^{\text{Ac}(H+)}$, b) $G_{1}^{\text{Ac}}$, c) $G_{2}^{\text{Ac}(H+)}$, and d) $G_{5}^{\text{Ac}}$. [Color figure can be viewed at wileyonlinelibrary.com]

Figure 6a presents a tertiary structure taken from the cluster $G_{1}^{\text{Ac}(H+)}$, where the aK382 and L383 side‐chains form a hydrophobic contact in the helix. Similarly, a conformation taken from $G_{1}^{\text{Ac}}$ shows that a hydrophobic contact is formed between aK382 and S378 (Fig. 6b). Figure 6c shows a hydrophobic contact between the side‐chain stems of aK382 and K381 in the partially helical conformation taken from $G_{2}^{\text{Ac}(H+)}$. Unless K382 is acetylated, a repulsive force acts between these two lysine residues. Furthermore, in this conformation, the oxygen atom in the aK382 side‐chain and the nitrogen atom in the K381 side‐chain interact electro‐statistically. Figure 6d presents a conformation taken from $G_{5}^{\text{Ac}}$, where a hydrophobic core is formed by side‐chain tips of aK382 and M384 and side‐chain stem of R379. Consequently, the acetylation induces the hydrophobic core formation in helices. Then, the radius of gyration becomes small. If Lys382 is nonacetyl form, then repulsion interactions take place between Lys382 and the other positively charged residues in the helix because each sequence of the fragments includes six or seven positively charged residues. Consequently, acetyl‐lysine stabilizes helical structures more than nonacetyl lysine does.

**Figure 7:**
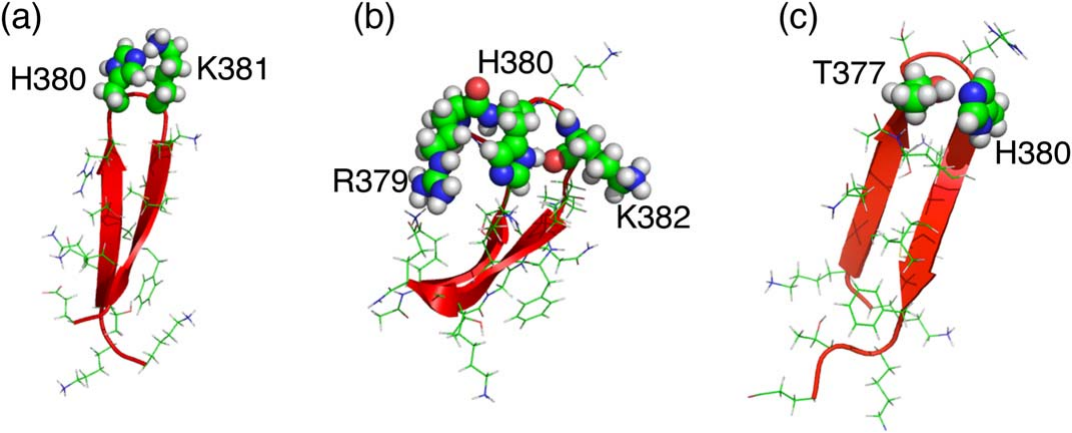
Tertiary structures taken from clusters a) $G_{4}^{\text{NonAc}}$, b) $G_{4}^{\text{NonAc}}$, and c) $G_{3}^{\text{Ac}}$. [Color figure can be viewed at wileyonlinelibrary.com]

As described above, comparison between Figures 3a and 3c as well as between Figures 3b and 3d clarified that the charge neutralization of H380 increases the probability in the broken‐line circled region, where hairpin structures are distributed. Figure 5 and Table 4 also show that the charge neutralization enhances the hairpin formation. Figure 7a portrays a hairpin taken from cluster $G_{4}^{\text{NonAc}}$, where H380 and K381 form a hydrogen bond. Repulsive interaction acts between H380 and K381 if H380 is positively charged. Similarly, Figure 7b displays a distorted hairpin taken from $G_{4}^{\text{NonAc}}$, where the side‐chain of H380 and main‐chain of K382 form a hydrogen bond. If H380 is positively charged, then this distorted hairpin becomes unstable because a repulsive interaction between the positively charged H380 and K382 might break the hydrogen bond. Furthermore, a repulsive interaction between the positively charged H380 and R379 might also destabilize the β‐hairpin structure. Consequently, the charge neutralization of H380 is necessary for stabilizing the hairpins in Figures 7a and 7b. Figure 7c displays a hairpin from $G_{3}^{\text{Ac}}$, where H380 and T377 form a hydrogen bond. These tertiary structures exemplify that the charge neutralized H380 serves the hydrogen bonds to stabilize turns in the hairpins.

Data shown in Figure 3 suggest visually that the Ac(H^+^) system might have the narrowest structural varieties among the four systems. To elucidate this feature quantitatively, we computed the standard deviation $\sigma_{\text{SYS}}^{}$ of the conformational distribution for Cα atoms for each system as

(10) $$\sigma_{\text{SYS}}^{}={\left[\sum_{i}\left(<q_{i}^{2}>-<q_{i}{>}^{2})\right]\right]}^{1/2},$$

where the brackets are the ensemble average over conformations in each ensemble $Q_{\text{SYS}}$ weighted at 300 K. The resultant values are: $\sigma_{\text{NonAc}(H+)}^{}=37.6$ Å, $\sigma_{\text{Ac}(H+)}^{}=30.6$ Å, $\sigma_{\text{NonAc}}^{}=36.5$ Å, and $\sigma_{\text{Ac}}^{}=36.9$ Å. These values of structural fluctuations are consistent with the radii of gyration in Table 3. Consequently, the Ac(H^+^) system has the smallest standard deviation. This reduction of the broadening results from the acetylation of K382. One might expect the Ac system to have a narrow distribution because K382 is also acetylated in this system. However, as shown above, the neutralization of H380 induces hairpins, which prevents reduction of the distribution.

A temperature replica exchange MD simulation^24^, ^26^ of a 14‐residue p53 CTD fragment and a conventional MD simulation^24^, ^26^ of a 15‐residue p53 CTD fragment were performed to obtain a conformational ensemble in the unbound state. These two fragments are fully included in our 17‐residue fragment, and H380 and K382 were positively charged, respectively, and K382 was nonacetylated. Consequently, those fragments are parts of the NonAc(H^+^) fragment, and binds to the S100B molecule with adoption of a helical conformation. It is particularly interesting that, in these studies, the ensembles involved a helical fraction, which is consistent with our result for $Q_{\text{NonAc}(H+)}$. In contrast, recent CD measurement of two 32‐residue p53 CTD fragments, which involve our NonAc and Ac segments, showed that the conformations of the CTD fragments are randomized.^48^ Our computational results demonstrated that both $Q_{\text{NonAc}}$ and $Q_{\text{Ac}}$ contain a helical fraction and that the helical content of $Q_{\text{Ac}}$ is larger than that of $Q_{\text{NonAc}}$. This apparent inconsistency between the computational results and the CD‐experimental observation should be analyzed. We note, however, that the fragment length for the CD experiment is considerably longer than ours.

**Figure 8:**
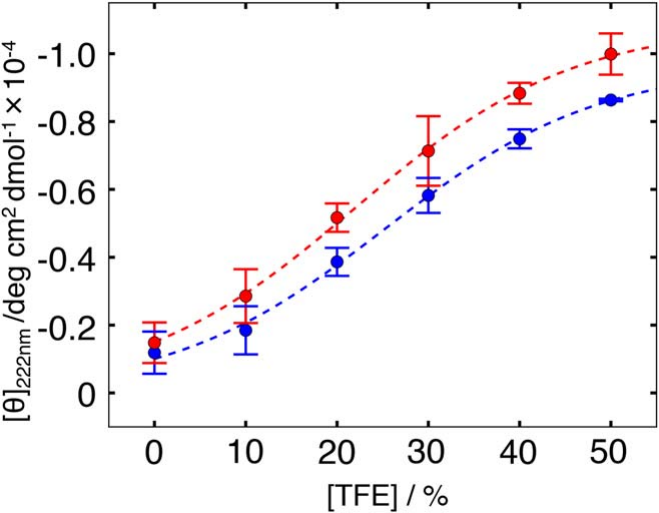
TFE‐concentration dependence of mean molar ellipticity per residue, [θ], at 222 nm at 25°C for NonAc and Ac fragments. Blue and red points, respectively, present θ for the NonAc and Ac fragments. The smaller the value of θ at a TFE concentration, the greater the helix content at the concentration. Blue and red broken lines, respectively, stand for fitting lines of the points of NonAc and Ac by the sigmoidal function. [Color figure can be viewed at wileyonlinelibrary.com]

To link the computation and experiment, we conducted CD experiments of the NonAc and Ac fragments of 17 residues long. TFE enhances formation of secondary structures, especially of helix.^49^, ^50^ To clarify the inherent helix propensity of the two fragments, the measurement was done at various TFE concentrations. CD spectra at zero TFE concentration have suggested that the overall structural feature of both fragments is characterized by a disorder state (data not shown), which is consistent to the preceding CD measurement.^48^ Therefore, the helical contents obtained from the simulations were larger than those from the CD experiments. In fact, although CD experiments are useful to discuss the secondary‐structure properties of polypeptide qualitatively, the CD data might involve quantitative ambiguity in assessing the secondary‐structure contents. Conversely, the simulation data might involve some errors. Therefore, we compare the simulation data with the CD data qualitatively. Figure 8 shows that the Ac fragment has a higher helix contents than the NonAc fragment at all examined TFE concentrations including the zero TFE concentration. This result was also supported by analysis of the CD spectra using “Bestsel” software^51^ (data not shown), which estimates the secondary‐structure contents from CD spectra. Therefore, we conclude from both computation and experimentation that the acetylation of K382 enhances the helix formation slightly.

As described earlier, NonAc(H^+^) binds to S100B with adopting helical conformation and Ac(H^+^) does not adopt a helical conformation to bind to a partner (Table 1). This experimentally obtained result might be inconsistent to the computational result that $Q_{\text{NonAc}(H+)}$ contains a smaller helix content than $Q_{\text{Ac}(H+)}$. We discuss possible binding mechanisms of the CTD fragments to their partner molecules in the next section.

### Free energy landscape of the common binding region

**Figure 9:**
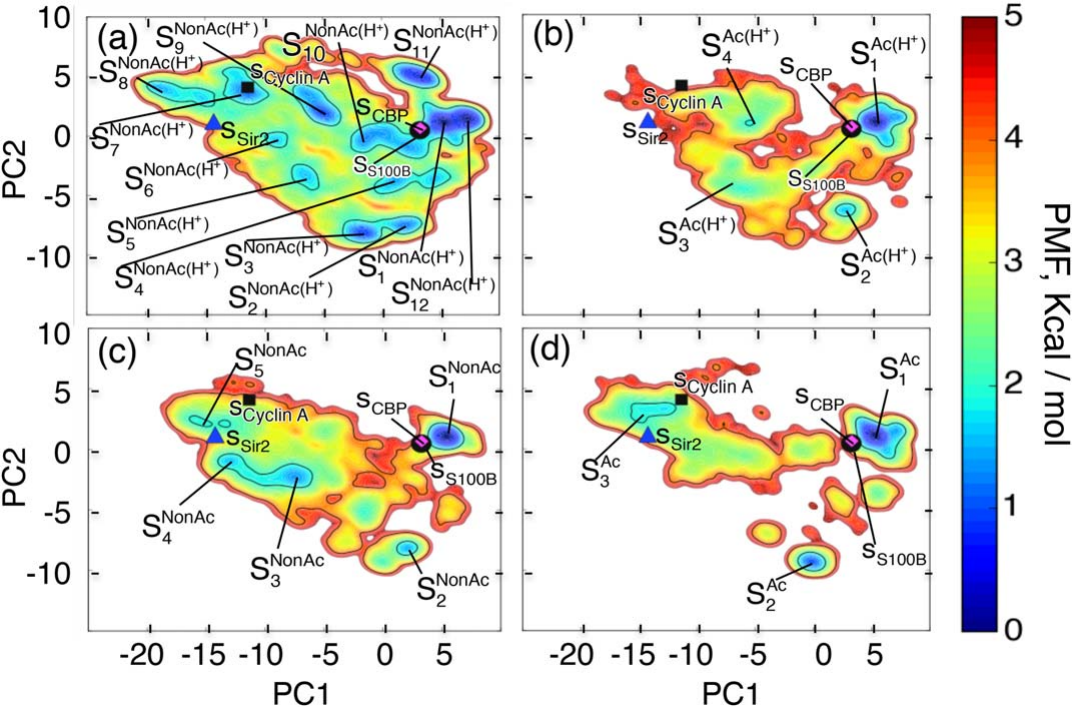
FEL, $F_{}^{\text{SYS}}$, at 300 K for the common binding region (residues 380–386) constructed in 2D PCA space: a) $F_{}^{\text{NonAc}(H+)}$, b) $F_{}^{\text{Ac}(H+)}$, c) $F_{}^{\text{NonAc}}$, and d) $F_{}^{\text{Ac}}$. The lowest PMF site, $r_{\text{min}}$, is set as $F_{}^{\text{SYS}}(r_{\text{min}})=0.0$ kcal/mol. Clusters $S_{k}^{\text{SYS}}$ are shown in FEL. Black filled circle, blue triangle, black filled square, and magenta colored diamond, respectively, denote positions of the bound forms $s_{S100B}$, $s_{\text{Sir}2}$, $s_{\text{Cyclin}}$, and $s_{\text{CBP}}$. Table 1 presents the correspondence between the CTD‐fragment type and the binding partner. PCA1 and PCA2 axes are computed from the entire ensemble (i.e., $Q_{\text{sum}}$). Consequently, the PCA axes for all panels are common. [Color figure can be viewed at wileyonlinelibrary.com]

As described in the *Introduction* section, the p53 CTD has a hub property. Four CTD‐partner complex structures were determined. As described in the *Materials and methods* section, the residues 380–386 of CTD are the common binding region to all the four partners S100B, Sir2, CBP, and Cyclin A. We computed the 2D FEL for this common binding region (Fig. 9), where the variance‐covariance matrix was computed only for the common binding region. This figure shows that various clusters are distributed in the 2D PCA space for all the ensembles. The contribution ratios are $rc_{1}=73.5$ % and $rc_{2}=14.4$ %; then $rc_{1}+rc_{2}=87.9$ %.

**Figure 10:**
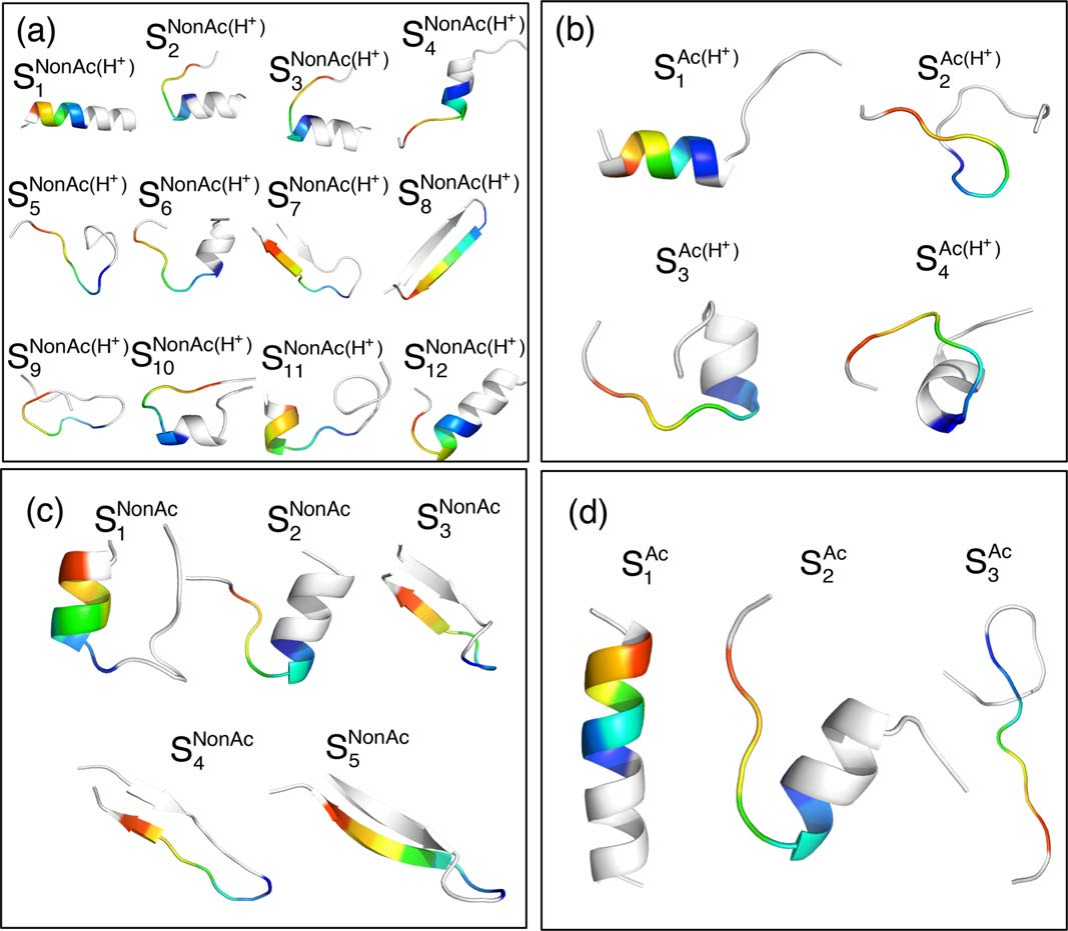
Structures in clusters of FEL for the common binding region: Tertiary structures picked from clusters $S_{k}^{\text{SYS}}$ in FEL are colored by rainbow for the common binding region (blue and red sides are, respectively, the N and C‐termini) and white for the other regions, which are not used for PCA. [Color figure can be viewed at wileyonlinelibrary.com]

We refer to the clusters as $S_{k}^{\text{SYS}}$, where superscript SYS specifies the computed system and subscript k is a label assigned to clusters in Figure 9. Cluster $S_{1}^{\text{SYS}}$ is assigned to the global minimum of PMF in all panels along with FEL for the full‐length fragments. Figure 10 presents representative tertiary structures in each cluster. Again, this cluster corresponds to a helical cluster (see structures in $S_{1}^{\text{SYS}}$ in Fig. 10) located at the same position in all panels.

Apparently, the ensemble $Q_{\text{NonAc}(H+)}$ has the broadest distribution of the four ensembles. Many clusters are found in FEL (Fig. 9a). The tertiary structures taken from the clusters are diverse (Fig. 10a): The complete helix is $S_{1}^{\text{NonAc}(H+)}$; partial helices are $S_{2}^{\text{NonAc}(H+)}$, $S_{3}^{\text{NonAc}(H+)}$, $S_{4}^{\text{NonAc}(H+)}$, $S_{6}^{\text{NonAc}(H+)}$, $S_{10}^{\text{NonAc}(H+)}$, $S_{11}^{\text{NonAc}(H+)}$, and $S_{12}^{\text{NonAc}(H+)}$; β hairpins are $S_{7}^{\text{NonAc}(H+)}$ and $S_{8}^{\text{NonAc}(H+)}$; and random‐coiled structures are $S_{5}^{\text{NonAc}(H+)}$ and $S_{9}^{\text{NonAc}(H+)}$. Inter‐cluster transitions can take place readily because free‐energy barriers among the clusters are low.

**Figure 11:**
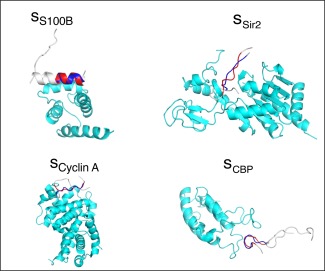
Experimentally determined complex structures and sampled conformations. The blue‐colored region is the common‐binding region of the experimental structure. Red is the sampled one. The sampled conformations are selected from the vicinity of the bound form in the free‐energy landscape (Fig. 9), and are superimposed on the bound form. White‐colored regions are outside the common‐binding regions. Cyan‐colored regions are partners. [Color figure can be viewed at wileyonlinelibrary.com]

Structural diversity decreases when K382 is acetylated and/or H380 is neutralized. In $Q_{\text{Ac}(H+)}$, no β hairpin cluster exists, although three partial helix clusters ( $S_{1}^{\text{Ac}(H+)}$, $S_{3}^{\text{Ac}(H+)}$_,_ and $S_{4}^{\text{Ac}(H+)}$) do (Fig. 9b). In $Q_{\text{NonAc}}$, only two helix clusters ( $S_{1}^{\text{NonAc}}$ and $S_{2}^{\text{NonAc}}$) and three β hairpin clusters ( $S_{3}^{\text{NonAc}}$, $S_{4}^{\text{NonAc}}$_,_ and $S_{5}^{\text{NonAc}}$) exist (Fig. 9c). In $Q_{\text{Ac}}$, two helix clusters ( $S_{1}^{\text{Ac}}$ and $S_{2}^{\text{Ac}}$) exist, but no hairpin cluster exists (Fig. 9d). In fact, the free‐energy barriers among clusters in Figures 9b–9d are higher than those in Figure 9a. Therefore, inter‐cluster transitions in Figures 9b–9d occur by passing narrower regions than those in Figure 9a. Figure 11 displays the experimentally determined complex structures and sampled conformations that are located near the bound form in the free‐energy landscape (Fig. 9). Apparently, the sampled conformations closely resemble the experimental bound form.

The bound forms of the common binding region in the experimentally determined complex structures are also assigned to Figure 9. Bound forms $s_{S100B}$ and $s_{\text{CBP}}$ are mutually close in Figure 9 although $s_{S100B}$ and $s_{\text{CBP}}$, respectively, denote a helix and a twisted conformation (See Fig. S1 of Supporting Information). The closeness of the two conformations results from the similarity of the Cα atomic pair distances. Remember that the PCA space is generated based on the inter‐Cα atomic distances. Therefore, $s_{\text{CBP}}$ can convert to $s_{S100B}$ by minor rearrangements of the Cα atomic pair distances.

Figure 9 proposes possible mechanisms of CTD binding to their partner molecules. We infer that the main binding mechanism of NonAc(H^+^) to S100B is the population selection because $s_{S100B}$ is located at a fringe of the most stable cluster $S_{1}^{\text{NonAc}(H+)}$ (Fig. 9a). In other words, $Q_{\text{NonAc}(H+)}$ prepares the bound form in advance. Furthermore, because the free‐energy barriers from the other clusters to $S_{1}^{\text{NonAc}(H+)}$ are low as described above, the bound form is recruited quickly when the bound form is exhausted to bind to S100B. The helical content of $Q_{\text{NonAc}(H+)}$ is smaller than that of $Q_{\text{Ac}(H+)}$. Figure 9a suggests that $Q_{\text{NonAc}(H+)}$ contains helical conformations sufficient for use for binding to S100B even if the helical content of $Q_{\text{NonAc}(H+)}$ is less than that of $Q_{\text{Ac}(H+)}$.

The population‐selection mechanism might take place when the Ac fragment binds to CBP, where the conformations in the most stable cluster $S_{1}^{\text{Ac}}$ can transition readily to the conformation $s_{\text{CBP}}$ (Fig. 9d). However, the cluster $S_{1}^{\text{Ac}}$ is not connected to the other clusters by low free‐energy pathways. Consequently, the recruitment of conformations to $S_{1}^{\text{Ac}}$ from the other clusters might be slow. In other words, the rate constant for the Ac fragment binding to CBP might be smaller than that for the NonAc(H^+^) binding to S100B if the population‐selection mechanism occurs.

For binding of the Ac(H^+^) fragment to Sir2, the conformation $s_{\text{Sir}2}$ is located at a high free‐energy site in Figure 9b. Therefore, we presume that the fragment binds to Sir2 with a different conformation than $s_{\text{Sir}2}$, by which an encounter complex is formed. Then, the intermolecular interactions bring the fragment to the genuine complex structure. Therefore, we presume that the binding mechanism of this fragment belongs to the induced folding mechanism.

The NonAc fragment can bind to either S100B or Cyclin A (Table 1). The binding mechanism to S100B might be the population selection for the same reason for the NonAc(H^+^) fragment binding to S100B. The structure $s_{\text{Cyclin}}$ is located at a site with free energy of about 3 kcal/mol in Figure 9c. Therefore, a small fraction of the ensemble $Q_{\text{NonAc}}$ is a conformation close to $s_{\text{Cyclin}}$. Consequently, the binding mechanism of this fragment to Cyclin A might belong to the population selection. However, the main fraction of $Q_{\text{NonAc}}$ is far from $s_{\text{Cyclin}}$. Therefore, different conformations might be used to bind to Cyclin A. Consequently, the induced folding mechanism is also possible.

It is likely that the diversity of FEL modulated by the state variation of lysine and/or histidine induces the hub property of CTD. One can reasonably infer that different FELs have different interaction mechanisms to other molecules. A single protein segment can have multiple binding partners. This property is called a hub. It is noteworthy that the flexibility of the CTD segment is fundamentally important for the diversity of FEL. If CTD is a structurally well‐defined portion of the protein, then FEL has no great diversity. Therefore, CTD might bind only to a single partner.

The binding mechanism proposed here is based only on the FEL of the unbound state, which means that no IDR–partner interactions are considered. To ascertain whether the proposed mechanism is correct or not, we should perform simulations of systems where CTDs and their partner molecules coexist, as in earlier studies.^11^, ^32^ However, the current study is useful to investigate the variation of FEL in the presence or absence of the partner.

Finally, we confirmed the convergence of the sampled data. As reported in the convergence of sampling–section in Supporting Information, the convergence is good for all the systems.

## Conclusions

To investigate a highly flexible biomolecular system, computational approaches are fundamentally important because experimental detection of large fluctuations at an atomistic resolution is still difficult. Because the high flexibility is an inherent property of IDR, investigation of the conformational ensemble is necessary to elucidate the nature of IDR. Therefore, a powerful conformational sampling method is required. We performed the enhanced conformational sampling method, V‐McMD, to obtain the conformational ensembles of four p53 CTD fragments in the unbound state at the atomic resolution in an explicit solvent. Then, we constructed free‐energy landscapes from the obtained conformational ensembles.

The shape of the free‐energy landscape varied depending on the K382 acetylation and/or the H380 neutralization in CTD. It is particularly interesting that acetylation enhanced the helix propensity. This computational result was confirmed using CD experiments. We also demonstrated that acetylation induces the hydrophobic‐core formation. The H380 neutralization has enhanced the hairpin formation of CTD. The helix content obtained from V‐McMD tends to be larger than that from the CD experiment. This fact suggests that the force field is imperfect and that there have not been accurate force fields yet.^52^ Results from the CD experiment were explained by the McMD simulation with atomistic details. Therefore, we believe that our results are useful to discuss the variation of CTD's conformational ensemble. Furthermore, the current results might assist in the generation of a general model for understanding the switching mechanism conducted by PTMs.

Each of the four CTD fragments has particular binding partner(s). We proposed possible binding mechanisms from the free‐energy landscape of the unbound state. To judge whether the proposed mechanisms are correct or not, sampling of systems consisting of CTD and their partners is necessary for the next stage of research. However, as discussed in the *Introduction*, the binding mechanism of IDR is determined not only by the finally formed complex structure but also by the conformational distribution in the single‐chain state. As discussed in *Results*, the spreading of the free‐energy landscape and the free‐energy barriers might affect the IDR‐partner binding mechanism. Therefore, results of the current study of the unbound state are expected to be useful to investigate the variation of the free‐energy landscape in the presence of the partner molecules. The results will provide useful knowledge to ascertain the hub property and coupled folding and binding of CTD.

## Supporting information

Supporting Information

## References

[1] V. N. Uversky , A. K. Dunker , Biochim. Biophys. Acta 2010, 1804, 1231. 20117254 10.1016/j.bbapap.2010.01.017 PMC2882790

[2] V. N. Uversky , C. J. Oldfield , A. K. Dunker , Annu. Rev. Biophys. 2008, 37, 215. 18573080 10.1146/annurev.biophys.37.032807.125924

[3] A. Patil , K. Kinoshita , H. Nakamura , Int. J. Mol. Sci. 2010, 11, 1930. 20480050 10.3390/ijms11041930 PMC2871146

[4] S. J. Metallo , Curr. Opin. Chem. Biol. 2010, 14, 481. 20598937 10.1016/j.cbpa.2010.06.169 PMC2918680

[5] F. Theillet , A. Binol , T. Frembgen‐kesner , K. Hingorani , M. Sarkar , C. Kyne , C. Li , P. B. Crowley , L. Gierasch , G. J. Pielak , A. H. Elcock , A. Gershenson , P. Selenko , Chem. Rev. 2015, 114, 6661. 10.1021/cr400695p PMC4095937 24901537

[6] R. B. Berlow , H. J. Dyson , P. E. Wright , FEBS Lett. 2015, 589, 2433. 26073260 10.1016/j.febslet.2015.06.003 PMC4586362

[7] K. Sugase , H. J. Dyson , P. E. Wright , Nature 2007, 447, 1021. 17522630 10.1038/nature05858

[8] J. Monod , J. Wyman , J. P. Changeux , J. Mol. Biol. 1965, 12, 88. 14343300 10.1016/s0022-2836(65)80285-6

[9] K. Okazaki , S. Takada , Proc. Natl. Acad. Sci. USA 2008, 105, 11182. 18678900 10.1073/pnas.0802524105 PMC2516237

[10] T. Yamane , H. Okamura , Y. Nishimura , A. Kidera , M. Ikeguchi , J. Am. Chem. Soc. 2010, 132, 12653. 20722414 10.1021/ja103218x

[11] J. Higo , Y. Nishimura , H. Nakamura , J. Am. Chem. Soc. 2011, 133, 10448. 21627111 10.1021/ja110338e

[12] P. Tompa , M. Fuxreiter , Trends Biochem. Sci. 2008, 33, 2. 18054235 10.1016/j.tibs.2007.10.003

[13] B. A. Shoemaker , J. J. Portman , P. G. Wolynes , Proc. Natl. Acad. Sci. USA 2000, 97, 8868. 10908673 10.1073/pnas.160259697 PMC16787

[14] C. J. Oldfield , J. Meng , J. Y. Yang , M. Q. Yang , V. N. Uversky , A. K. Dunker , BMC Genomics 2008, 9 Suppl 1, S1. 10.1186/1471-2164-9-S1-S1 PMC2386051 18366598

[15] E. Bochkareva , L. Kaustov , A. Ayed , G. Yi , Y. Lu , A. Pineda‐Lucena , J. C. C. Liao , A. L. Okorokov , J. Milner , C. H. Arrowsmith , A. Bochkarev , Proc. Natl. Acad. Sci. USA 2005, 102, 15412. 16234232 10.1073/pnas.0504614102 PMC1266094

[16] P. Di Lello , L. M. M. Jenkins , T. N. Jones , B. D. Nguyen , T. Hara , H. Yamaguchi , J. D. Dikeakos , E. Appella , P. Legault , J. G. Omichinski , Mol. Cell 2006, 22, 731. 16793543 10.1016/j.molcel.2006.05.007

[17] P. H. Kussie , S. Gorina , V. Marechal , B. Elenbaas , J. Moreau , A. J. Levine , N. P. Pavletich , Science 1996, 274, 948. 8875929 10.1126/science.274.5289.948

[18] R. Rust , D. Baldisseri , D. Weber , Nat. Struct. Mol. 2000, 7, 570. 10.1038/76797 10876243

[19] J. L. Avalos , I. Celic , S. Muhammad , M. S. Cosgrove , J. D. Boeke , C. Wolberger , Mol. Cell 2002, 10, 523. 12408821 10.1016/s1097-2765(02)00628-7

[20] S. Mujtaba , Y. He , L. Zeng , S. Yan , O. Plotnikova , Sachchidanand, R. Sanchez , N. J. Zeleznik‐Le , Z. Ronai , M.‐M. Zhou , Mol. Cell 2004, 13, 251. 14759370 10.1016/s1097-2765(03)00528-8

[21] E. D. Lowe , I. Tews , K. Y. Cheng , N. R. Brown , S. Gul , M. E. M. Noble , S. J. Gamblin , L. N. Johnson , Biochemistry 2002, 41, 15625. 12501191 10.1021/bi0268910

[22] J. van Dieck , D. P. Teufel , A. M. Jaulent , M. R. Fernandez‐Fernandez , T. J. Rutherford , A. Wyslouch‐Cieszynska , A. R. Fersht , J. Mol. Biol. 2009, 394, 922. 19819244 10.1016/j.jmb.2009.10.002

[23] W. J. Allen , D. G. S. Capelluto , C. V. Finkielstein , D. R. Bevan , J. Phys. Chem. B 2010, 114, 13201. 20873738 10.1021/jp1011445

[24] J. Chen , J. Am. Chem. Soc. 2009, 131, 2088. 19216110 10.1021/ja809547p

[25] C. McDowell , J. Chen , J. Chen , J. Mol. Biol. 2013, 425, 999. 23313430 10.1016/j.jmb.2013.01.001

[26] I. Staneva , Y. Huang , Z. Liu , S. Wallin , PLoS Comput. Biol. 2012, 8, e1002682. 23028280 10.1371/journal.pcbi.1002682 PMC3441455

[27] B. A. Berg , T. Neuhaus , Phys. Rev. Lett. 1992, 68, 9. 10045099 10.1103/PhysRevLett.68.9

[28] U. H. E. Hansmann , Y. Okamoto , F. Eisenmenger , Chem. Phys. Lett. 1996, 259, 321.

[29] N. Nakajima , H. Nakamura , A. Kidera , J. Phys. Chem. B 1997, 101, 817.

[30] T. Sugihara , J. Higo , H. Nakamura , J. Phys. Soc. Japan 2009, 78, 074003.

[31] J. Ikebe , K. Umezawa , N. Kamiya , T. Sugihara , Y. Yonezawa , Y. Takano , H. Nakamura , J. Higo , J. Comput. Chem. 2011, 32, 1286. 21425286 10.1002/jcc.21710

[32] K. Umezawa , J. Ikebe , M. Takano , H. Nakamura , J. Higo , Biomolecules 2012, 2, 104. 24970129 10.3390/biom2010104 PMC4030872

[33] J. Higo , K. Umezawa , H. Nakamura , J. Chem. Phys. 2013, 138, 184106. 23676028 10.1063/1.4803468

[34] T. Terakawa , J. Higo , S. Takada , Biophys. J. 2014, 107, 721. 25099811 10.1016/j.bpj.2014.06.026 PMC4129485

[35] The UniProt Consortium Nucleic Acids Res. 2014, 43, D204. 25348405 10.1093/nar/gku989 PMC4384041

[36] T. Mashimo , Y. Fukunishi , N. Kamiya , Y. Takano , I. Fukuda , H. Nakamura , J. Chem. Theory Comput. 2013, 9, 5599. 26592294 10.1021/ct400342e

[37] J. Ryckaert , G. Ciccotti , H. Berendsen , J. Comput. Phys. 1977, 327.341.

[38] I. Fukuda , N. Kamiya , Y. Yonezawa , H. Nakamura , J. Chem. Phys. 2012, 137, 054314. 22894355 10.1063/1.4739789

[39] I. Fukuda , Y. Yonezawa , H. Nakamura , J. Chem. Phys. 2011, 134, 164107. 21528950 10.1063/1.3582791

[40] N. Kamiya , I. Fukuda , H. Nakamura , Chem. Phys. Lett. 2013, 568–569, 26.

[41] L. V. Woodcock , Chem. Phys. Lett. 1971, 10, 257.

[42] W. L. Jorgensen , J. Chandrasekhar , J. D. Madura , R. W. Impey , M. L. Klein , J. Chem. Phys. 1983, 79, 926.

[43] N. Kamiya , Y. S. Watanabe , S. Ono , J. Higo , Chem. Phys. Lett. 2005, 401, 312.

[44] W. D. Cornell , P. Cieplak , C. I. Bayly , I. R. Gould , K. M. Merz , D. M. Ferguson , D. C. Spellmeyer , T. Fox , J. W. Caldwell , P. A. Kollman , J. Am. Chem. Soc. 1995, 117, 5179.

[45] P. Kollman , R. Dixon , W. Cornell , T. Fox , C. Chipot , A. Pohorille , In The Development/Application of a “Minimalist” Organic/Biochemical Molecular Mechanic Force Field Using a Combination of Ab Initio Calculations and Experimental Data, Vol. 3; van Gunsteren W., Weiner P., Wilkinson A., Eds.; Springer: Netherlands, 1997.

[46] J. Ikebe , N. Kamiya , J. Ito , H. Shindo , 2007, 16, 1596. 10.1110/ps.062721907 PMC2203368 17656579

[47] R. P. Joosten , T. A H. Te Beek , E. Krieger , M. L. Hekkelman , R. W. W. Hooft , R. Schneider , C. Sander , G. Vriend , Nucleic Acids Res. 2011, 39, 1. 21071423 10.1093/nar/gkq1105 PMC3013697

[48] O. D. Shahar , R. Gabizon , O. Feine , R. Alhadeff , A. Ganoth , L. Argaman , E. Shimshoni , A. Friedler , M. Goldberg , PLoS One 2013, 8, e78472. 24194938 10.1371/journal.pone.0078472 PMC3806821

[49] P. Luo , R. L. Baldwin , Biochemistry 1997, 36, 8413. 9204889 10.1021/bi9707133

[50] N. Hirota , K. Mizuno , Y. Goto , J. Mol. Biol. 1998, 275, 365. 9466915 10.1006/jmbi.1997.1468

[51] A. Micsonai , F. Wien , L. Kernya , Y. H. Lee , Y. Goto , M. Réfrégiers , J. Kardos , Proc. Natl. Acad. Sci. USA 2015, 112, E3095. 26038575 10.1073/pnas.1500851112 PMC4475991

[52] K. Lindorff‐Larsen , P. Maragakis , S. Piana , M. P. Eastwood , R. O. Dror , D. E. Shaw , Plos One 2012, 7, 1. 10.1371/journal.pone.0032131 PMC3285199 22384157

