# Supplementary material for: Variation of free‐energy landscape of the p53 C‐terminal domain induced by acetylation: Enhanced conformational sampling
Source: J Comput Chem. 2016 Oct 13;37(31):2687–700. doi: 10.1002/jcc.24494 (PMC5242334; doi:10.1002/jcc.24494)
Supplement: Supplementary file 1 — Supporting Information [file JCC-37-2687-s001.docx]

**Supplementary Materials**

**Variation of Free-Energy Landscape of the p53 C-terminal Domain Induced by Acetylation: Enhanced Conformational Sampling**

Shinji Iida,^1^ Tadaaki Mashimo,^2,3^ Takashi Kurosawa,^3,4^ Hironobu Hojo,^1^ Hiroya Muta,^1^ Yuji Goto,^1^ Yoshifumi Fukunishi,^4,5^ Haruki Nakamura,^1^ Junichi Higo^1^

1 Institute for Protein Research, Osaka University, Suita, Osaka 565-0871, Japan.

2 Technology research association for next generation natural products chemistry, 2-3-26 Aomi, Koto-Ku, Tokyo, 135-0064, Japan.

3 IMSBIO Co., Ltd. Owl tower 6F, 4-21-1, Higashi-ikebukuro, Toshima-ku, Tokyo 170-0013 JAPAN

4 Hitachi Solutions East Japan, 21-1 Ekimaehoncho, Kawasaki-ku, Kanagawa 210-0007, Japan.

5 Molecular Profiling Research Center for Drug Discovery (molprof), National Institute of advanced Industrial Sciemce and Technology, (AIST), 2-3-36, Aomi, Koto-ku, Tokyo 135-0064, Japan.

**Table S1**. Parameters for V-McMD. ^a^

----------------------------------------------------------------------------------------------------------------------------------

Iteration No. $N_{\text{int}}$ ^b^ Simulation length ^c^ Zone ^d^

----------------------------------------------------------------------------------------------------------------------------------

#1 20000 192 [0.0, 0.1], [0.05, 0.2], [0.1, 0.3], [0.2, 0.45], [0.3, 0.6],

[0.45, 0.8], [0.6, 1.0]

#2 Same as #1 192 Same as #1

#3 Same as #1 192 Same as #1

#4 Same as #1 192 Same as #1

#5 Same as #1 192 Same as #1

#6 Same as #1 192 Same as #1

#7 10000 192 Same as #1

#8 Same as #7 192 [0.0, 0.1], [0.05, 0.2], [0.1, 0.3], [0.2, 0.45], [0.3, 0.6],

[0.45, 1.0]

#9 ^e^ Same as #8 320 Same as #8

----------------------------------------------------------------------------------------------------------------------------------

^a^ Shown parameters are those from the Ac(H^+^) system. Data for the other systems are slightly different.

^b^ $N_{\mathrm{int}}$ is inter-virtual state transition interval. Unit is steps.

^c^ Shown values are summation of simulation lengths of 32 multiple runs, and value multiplied by $10^{6}$ is the simulation length. Unit is ns.

^d^ A bracket corresponds to zone $[E_{\text{R}}^{\text{min}}(v_{i}),E_{\text{R}}^{\text{max}}(v_{i})]$ for virtual state $v_{i}$, and is given in a normalized form as $[\lambda_{1},\lambda_{2}]$ ($0\leq\lambda_{1}<\lambda_{2}\leq1$): $E_{\text{R}}^{\text{min}}=\lambda_{1}\Delta E+E_{\text{low}}$ and $E_{\text{R}}^{\text{max}}=\lambda_{2}\Delta E+E_{\text{low}}$, where $\Delta E=E_{\text{up}}-E_{\text{low}}$. $E_{\text{up}}$ and $E_{\text{low}}$ are the maximum and minimum energies over all virtual states: $E_{\text{up}}=-32780.0$ kcal / mol and $E_{\text{low}}=-42760.0$ kcal / mol. Zones are listed as $[E_{\text{R}}^{\text{min}}(v_{1}),E_{\text{R}}^{\text{max}}(v_{1})]$, $[E_{\text{R}}^{\text{min}}(v_{2}),E_{\text{R}}^{\text{max}}(v_{2})]$, and so on.

^e^ Iteration 9 is production run.


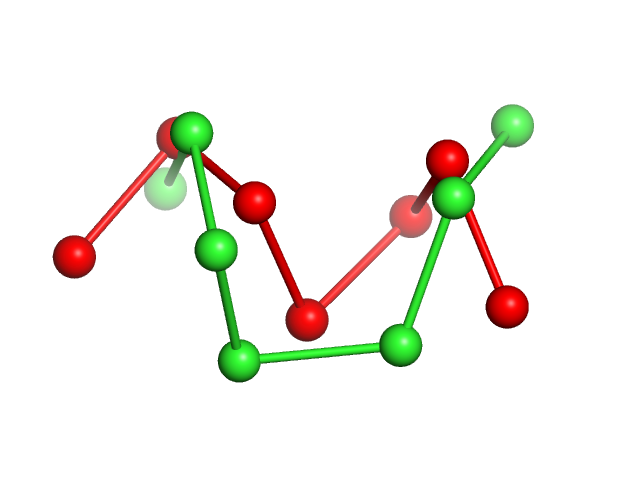


**Figure S1**. Cα atomic trace of bound conformations $\text{s}_{\text{S100B}}$ (red colored model) and $\text{s}_{\text{CBP}}$ (green colored model) of common binding regions (residues 380-386). See figure captions of Figure 9 for $\text{s}_{\text{S100B}}$ and $\text{s}_{\text{CBP}}$ in the main text.

**Convergence of sampling**

As shown in the main body, the V-McMD produced flat distributions (Figure 2 in the main text), which ensures that the sampled conformations have statistically significance in the wide energy range from 280 K to 600 K. Here we further checked statistical significance of the resultant ensembles $\text{Q}_{\text{SYS}}$ by analyzing the 2D FEL (i.e., $F^{\text{SYS}}$) as follows: We divided the trajectories into halves, where the trajectory ensembles consisting of the former and latter halves are denoted as $\text{H}_{1}$ and $\text{H}_{2}$, respectively. We express the FEL value at a 2D site $r_{m}$ computed from $\text{H}_{i}$ as $F_{\text{H}i}^{\text{SYS}}(r_{m})$, where $i=1,2$. Figure S2a is 2D FEL computed from $\text{H}_{1}$, and Figure S2b is that from $\text{H}_{2}$ for the common binding region of the Ac system, which shows that the patterns of FELs are similar to each other. For quantitative assessment, a correlation coefficient $C_{\text{cor}}$ between the two FELs is defined as:

$C_{\text{cor}}^{\text{SYS}}=\frac{\left\langle F_{\text{H1}}^{\text{SYS}}F_{\text{H2}}^{\text{SYS}}\right\rangle-\left\langle F_{\text{H1}}^{\text{SYS}}\right\rangle\left\langle F_{\text{H2}}^{\text{SYS}}\right\rangle}{\sigma_{\text{H}1}^{\text{SYS}}\sigma_{\text{H}2}^{\text{SYS}}}$, (S1)

where

$\left\langle F_{\text{H}i}^{\text{SYS}}F_{\text{H}j}^{\text{SYS}}\right\rangle=\frac{1}{N}\sum_{m}^{N\text{site}} F_{\text{H}i}^{\text{SYS}}(r_{m})F_{\text{H}j}^{\text{SYS}}(r_{m}) (i=1,2 \text{and} j=1,2)$ (S2)

$\left\langle F_{\text{H}k}^{\text{SYS}}\right\rangle=\frac{1}{N}\sum_{m}^{N\text{site}} F_{\text{H}k}^{\text{SYS}}(r_{m}) (k=1,2)$ (S3)

$\sigma_{\text{H}k}^{\text{SYS}}=\left[\left\langle(F_{\text{H}k}^{\text{SYS}})^{2}\right\rangle-\left\langle F_{\text{H}k}^{\text{SYS}}\right\rangle^{2}\right]^{1/2} (k=1,2)$ (S4)

The summations in the above equations are taken over sites that satisfy $F_{\text{H}i}^{\text{SYS}}(r_{m})\leq3.0$ in both of $F_{\text{H}1}^{\text{SYS}}(r_{m})$ and $F_{\text{H}2}^{\text{SYS}}(r_{m})$ to focus on well-sampled 2D PCA regions, and $N_{\text{site}}$ is the number of those sites. We demonstrate correlation coefficient of each *F*^SYS^ for the common binding region: The highest correlation coefficient was obtained from the Ac system ($C_{\text{cor}}^{\text{Ac}}=0.76$) and the lowest was from the NonAc(H^+^) system ($C_{\text{cor}}^{\text{NonAc(H+)}}=0.45$). The intermediate correlations are: $C_{\text{cor}}^{\text{NonAc}}=0.60$ from the NonAc system and $C_{\text{cor}}^{\text{Ac(H+)}}=0.53$ from the Ac(H^+^) system. Therefore, the convergence is relatively well for all of the systems. When we computed the correlation coefficient with taking sites with high PMF values, the correlation coefficient became small (data not shown).

**Figure S2**. 2D PMFs $F_{\text{H1}}^{\text{SYS}}$ and $F_{\text{H2}}^{\text{SYS}}$ at 300 K computed from ensembles $\text{H}_{1}$ (a) and $\text{H}_{2}$ (b) of the common binding region of the Ac system, respectively.
